# *BRCA1* mutations and other sequence variants in a population-based sample of Australian women with breast cancer

**DOI:** 10.1038/sj.bjc.6690008

**Published:** 1999-09-24

**Authors:** M C Southey, A A Tesoriero, C R Andersen, K M Jennings, S M Brown, G S Dite, M A Jenkins, R H Osborne, J A Maskiell, L Porter, G G Giles, M R E McCredie, J L Hopper, D J Venter

**Affiliations:** Department of Pathology and Research, Peter MacCallum Cancer Institute, Victoria 3000, Australia; Department of Pathology, The University of Melbourne, Parkville, Victoria 3052, Australia; The University of Melbourne, Genetic Epidemiology Unit, Carlton, Victoria 3053, Australia; Cancer and Epidemiology Research Unit, New South Wales Cancer Council, Woolloomooloo, NSW 2011, Australia; Cancer Epidemiology Centre, The Anti-Cancer Council of Victoria, Carlton, Victoria 3053, Australia; Department of Preventive and Social Medicine, University of Otago, Dunedin, New Zealand

**Keywords:** *BRCA1*, breast cancer, DNA sequencing, mutations, population prevalence, variants

## Abstract

The frequency, in women with breast cancer, of mutations and other variants in the susceptibility gene, *BRCA1*, was investigated using a population-based case–control-family study. Cases were women living in Melbourne or Sydney, Australia, with histologically confirmed, first primary, invasive breast cancer, diagnosed before the age of 40 years, recorded on the state Cancer Registries. Controls were women without breast cancer, frequency-matched for age, randomly selected from electoral rolls. Full manual sequencing of the coding region of *BRCA1* was conducted in a randomly stratified sample of 91 cases; 47 with, and 44 without, a family history of breast cancer in a first- or second-degree relative. All detected variants were tested in a random sample of 67 controls. Three cases with a (protein-truncating) mutation were detected. Only one case had a family history; her mother had breast cancer, but did not carry the mutation. The proportion of Australian women with breast cancer before age 40 who carry a germline mutation in *BRCA1* was estimated to be 3.8% (95% Cl 0.3–12.6%). Seven rare variants were also detected, but for none was there evidence of a strong effect on breast cancer susceptibility. Therefore, on a population basis, rare variants are likely to contribute little to breast cancer incidence. © 1999 Cancer Research Campaign


*British Journal of Cancer* (1999) **79**(1), 34–39
© 1999 Cancer Research Campaign

### *BRCA1* mutations and other sequence variants in a population-based sample of Australian women with breast cancer

**MC Southey[1,2], AA Tesoriero[1], CR Andersen[1], KM Jennings[1], SM Brown[1], GS Dite[3], MA Jenkins[3], RH Osborne[3], JA Maskiell[3], L Porter[4], GG Giles[5], MRE McCredie[4,6], JL Hopper[3] and DJ Venter[1,2]**

[1]Department of Pathology and Research, Peter MacCallum Cancer Institute, Melbourne, Victoria 3000, Australia; [2]Department of Pathology, The University of Melbourne, Parkville, Victoria 3052, Australia; [3]The University of Melbourne, Genetic Epidemiology Unit, 200 Berkeley Street, Carlton, Victoria 3053, Australia; [4]Cancer and Epidemiology Research Unit, New South Wales Cancer Council, Woolloomooloo, NSW 2011, Australia; [5]Cancer Epidemiology Centre, The Anti-Cancer Council of Victoria, Carlton, Victoria 3053, Australia; [6]Department of Preventive and Social Medicine, University of Otago, Dunedin, New Zealand

**Summary** The frequency, in women with breast cancer, of mutations and other variants in the susceptibility gene, *BRCA1*, was investigated using a population-based case–control-family study. Cases were women living in Melbourne or Sydney, Australia, with histologically confirmed, first primary, invasive breast cancer, diagnosed before the age of 40 years, recorded on the state Cancer Registries. Controls were women without breast cancer, frequency-matched for age, randomly selected from electoral rolls. Full manual sequencing of the coding region of *BRCA1* was conducted in a randomly stratified sample of 91 cases; 47 with, and 44 without, a family history of breast cancer in a first- or second-degree relative. All detected variants were tested in a random sample of 67 controls. Three cases with a (protein-truncating) mutation were detected. Only one case had a family history; her mother had breast cancer, but did not carry the mutation. The proportion of Australian women with breast cancer before age 40 who carry a germline mutation in *BRCA1* was estimated to be 3.8% (95% CI 0.3–12.6%). Seven rare variants were also detected, but for none was there evidence of a strong effect on breast cancer susceptibility. Therefore, on a population basis, rare variants are likely to contribute little to breast cancer incidence.

**Keywords:** *BRCA1*; breast cancer; DNA sequencing; mutations; population prevalence; variants

Since the cloning of *BRCA1* and *BRCA2* (Miki et al, 1994; Wooster et al, 1995), there has been considerable interest in the population prevalence (allele frequency) and the age-specific cumulative risk (penetrance) of mutations in these genes, and the proportion of breast and other cancers attributable to these mutations (population attributable risk or aetiological fraction). To date, however, most information on the magnitude of the above population characteristics of these genes has been indirect, not based on population samples, or incomplete.

Indirect information on population characteristics has been derived from segregation analyses of nuclear families ascertained in case–control studies, in which the assessment of disease in relatives has been based on unverified reports from cases alone and usually restricted to first-degree relatives. The main purpose of segregation analysis is to elucidate the most likely mode of inheritance. Estimates are strongly dependent on sampling, and on the assumptions of the underlying mathematical model which usually attribute all of the familial aggregation of disease to genetic factors. Because at least a proportion of the familial aggregation of breast cancer can be explained by familial aggregation in epidemiological risk factors, especially taking into account misclassification and measurement error (Hopper and Carlin, 1992), segregation analyses are likely to overestimate any genetic contribution.

Analysis of data from US nuclear families ascertained in a case–control study of breast cancer predicted that about 1 in 150 women (confidence interval not reported) had inherited a lifetime risk of about 90% (Claus et al, 1991). A more recent analysis of data from US families ascertained in case–control studies of ovarian cancer suggested an allele frequency of about 1 in 350 [95% confidence interval (CI) from 1 in 50 to 1 in 2500] (Whittemore et al, 1997). Although it is sometimes presumed that these estimates are fully attributable to *BRCA1* (e.g. Whittemore et al, 1997), they should be interpreted as representing the dominantly inherited component of all genes involved in susceptibility to breast cancer, including *BRCA2*. Analysis of population-based samples of UK cancer families (Easton et al, 1996; Peto et al, 1996), assuming *BRCA1* accounts for almost all the excess of ovarian cancer in relatives of breast cancer patients and vice versa, estimated that 1 in 800 women (95% CI, 1 in 500 to 1 in 2500) inherit a *BRCA1* mutation (Ford et al, 1995).

In this paper, we estimate, among those who develop breast cancer before the age of 40 years, the proportion of women who carry a mutation in *BRCA1*. Indirect information from mathematical modelling initially suggested that about 30% of such early-onset breast cancer cases could occur in women who carry a high-risk dominantly inherited susceptibility (Claus et al, 1991), although recent publications have suggested that this may be more in the order of 11% (95% CI 1–50%) (Whittemore et al, 1997), or 5% (CI, not reported) (Ford et al, 1995). It could also vary from population to population.

*Received 4 February 1998*
*Revised 7 May 1998*
*Accepted 12 May 1998*

*Correspondence to:* JL Hopper, The University of Melbourne, Genetic Epidemiology Unit, 200 Berkeley Street, Carlton, Victoria 3053, Australia

**Table 1** *BRCA1* exon 11 primer sequences

| Primer name | PCR primer sequence | Sequencing primer | Region analysed[b] |
|---|---|---|---|
| 11.1 5′ | GGA ATT AAA TGA AAG AGT ATG AGC[a] | 5′ | 789–1140 |
| 11.1 3′ | CTC ACA CAG GGG ATC AGC ATT C[a] | | |
| 11.2 5′ | TGA ACA CCA CTG AGA AGC GTG | 3′ | 1021–1207 |
| 11.2 3′ | GAC ATT CCA AGA CTA CTG AGT GT | | |
| 11.3 5′ | CAA CAT AAC AGA TGG GCT GGA AG[a] | 5′ | 1129–1411 |
| 11.3 3′ | GCC AGT AAG TCT ATT TTC TCT GAA GAA C[a] | | |
| 11.4 5′ | GGT TCT GAT GAC TCA CAT GAT GGG[a] | 5′ | 1363–1590 |
| 11.4 3′ | TGT GAG GGG ACG CTC TTG | | |
| 11.5 5′ | TTG GGA AAA CCT ATC GGA A | 5′ | 1561–1796 |
| 11.5 3′ | CCA TGA GTT GTA GGT TTC TGC TG[a] | | |
| 11.6 5′ | ATC AGG GAA CTA ACC AAA CGG AG[a] | 5′ | 1790–2059 |
| 11.6 3′ | CCA TGA GTT GTA GGT TTC TGC TG[a] | | |
| 11.7 5′ | AGG CTG AGG AGG AAG TCT TCT ACC[a] | 5′ | 1996–2293 |
| 11.7 3′ | CCT GAG TGC CAT AAT CAG TAC CAG G[a] | 3′ | 2165–2404 |
| 11.8 5′ | GTG TTC AAA TAC CAG TGA ACT TA | 5′ | 2368–2638 |
| 11.8 3′ | TGT TCA CAT TCA AAA GTG | 3′ | 2598–2777 |
| 11.9 5′ | GCC AGT CAT TTG CTC CGT TTC[a] | 5′ | 2768–3050 |
| 11.9 3′ | GGA GCC CAC TTC ATT AGT AC | 3′ | 2964–3234 |
| 11.10 5′ | CCA AGT ACA GTG AGC ACA ATT A | 5′ | 3229–3420 |
| 11.10 3′ | CAG GAT GCT TAC AAT TAC TTC CAG G[a] | | |
| 11.11 5′ | TTG AAT GCT ATG CTT AGA TTA GGG G[a] | 5′ | 3417–3761 |
| 11.11 3′ | GTG ATG TTC CTG AGA TGC CTT TG[a] | | |
| 11.12 5′ | GAG TCC TAG CCC TTT CAC CCA TAC[a] | 5′ | 3744–4123 |
| 11.12 3′ | GTG CTC CCA AAA GCA TAC A[a] | 3′ | 3865–4215 |

[a]Primers described by Friedman et al (1994). [b]Sequence as per Genbank Accession No. U14680.

The first direct evidence came from mutation screening in 80 women from a previous US case–control study of breast cancer in women under the age of 35 (Langston et al, 1996), which found that 7.5% (95% CI 3.8–11.4%) carried ‘definite’ germline mutations. The sensitivity of the mutation detection method was considered to be 70–80%, and although blood was available from only one-third of eligible cases these did not appear to differ in frequency of family history from the non-tested, interviewed cases. A small proportion of carcinoma in situ was included, but all six mutation carriers had invasive breast cancer. Four rare variants of unknown significance were also observed. Interestingly, only three of the six mutation carriers, and one of the four carriers of a rare variant, reported breast cancer in a first- or second-degree female relative. More recently, mutation testing in another US population-based sample of 211 cases, over-sampled for onset before the age of 50 years, found that only three carried a mutation (all protein-truncating), leading to a prevalence estimate of 3.3% (95% CI 0–7.2%) among white women and 0% among African–American women (Newman et al, 1998).

We have conducted a full sequence analysis of the *BRCA1* coding region (and of some non-coding regions) in a population-based sample of women with breast cancer, stratified by family history, and in a random, population-based sample of women without breast cancer. Information on family history was obtained by interviewing cases, controls and relatives, and validated where possible. The mutation-detection approach we have used should detect all mutations in the coding and flanking intronic regions, including single base changes, but would not detect splicing problems due to genetic variation deep within the intronic regions or variants in the promotor region or outside *BRCA1*.

### SUBJECTS AND METHODS

A population-based case–control-family study of early onset breast cancer was carried out in Melbourne and Sydney from 1992 to 1995 (Hopper et al, 1994; McCredie et al, 1998). Cases were adult women under the age of 40 years at diagnosis of an incident, histologically confirmed, first primary, invasive breast cancer (ICD-9 174) identified through the Victorian and New South Wales state cancer registries. Controls were women who had not had breast cancer, selected from the electoral rolls (enrolment is compulsory in Australia) using stratified random sampling and frequency matched for age. As well as cases and controls, living relatives were interviewed face to face or by telephone.

For each proband (case or control), a detailed family history was systematically recorded for first- and second-degree relatives, and subsequently checked with living relatives at interview. For the purpose of sampling cases for mutation screening, individuals who reported at least one first- or second-degree female relative with breast cancer were considered to have a ‘family history’. Verification of every cancer reported in a family by either probands or relatives was sought through cancer registries, pathology reports, hospital records, treating clinicians and death certificates. Blood samples were collected from cases and controls, and from selected relatives in families with a history of cancer. A total of 467 cases (response rate 73%) and 408 controls (64%) were studied (McCredie et al, 1998). Of these, blood samples were available from 388 cases (60% of all eligible cases) and 294 controls. There were no differences between cases from whom blood was, or was not, collected in terms of age, country of birth, or any of the measured risk factors (Southey et al, 1998).

© Cancer Research Campaign 1999 

**Table 2** Presence or absence of a *BRCA1* protein-truncating mutation in women with breast cancer diagnosed before the age of 40 years, by family history status

| *BRCA1* mutation status | Family history[a] | | |
|---|---|---|---|
| | Yes | No | Total |
| Yes | 1 | 2 | 3 |
| No | 46 | 42 | 88 |
| Total | 47 | 44 | 91 |

[a]Family history is defined by at least one female first- or second-degree relative reported to have had breast cancer.

Two groups of cases were chosen for *BRCA1* sequencing by random stratified sampling: 47 who reported a family history of breast cancer, of whom we were able to verify 36 (77%), and 44 who did not report a family history. Variants identified in cases were tested for in a random sample of 67 controls.

The study was approved by the Institutional Ethics Committees of The University of Melbourne, The Anti-Cancer Council of Victoria, and the New South Wales Cancer Council.

### DNA preparation

DNA was extracted from stored buffy coat using a Progenome II DNA extraction kit (Progen, Australia) and stored in TE buffer (10 mM Tris. HCl/pH 8.0, 1 mM EDTA).

### PCR analysis

Each coding exon of *BRCA1* (except exon 11) was amplified using intronic primers based on those described by Simard et al (1994), located 5′ and 3′ to each exon. Exon 11 was amplified in twelve overlapping PCR fragments. The combinations of oligonucleotides and sequencing strategy necessary to sequence exon 11 are indicated in Table 1. Exon 7 was also amplified using the above intronic 5′ primer and a newly designed intronic 3′ primer (5′-GGC CAT GGT GCG CGT GCC GTG T -3′) [replacing the exonic primer described previously (Simard et al, 1994)]. Typical PCR reactions contained [10 ng DNA, 1× reaction buffer (Perkin Elmer), 1.5–2.5 mM magnesium chloride, 0.2 μM each PCR primer, 0.1 mM dNTPs and 0.5 units Amplitaq DNA polymerase (Perkin Elmer) in a final volume of 25 μl]. PCR fragments were amplified in a 96-well format in a Gene Amp PCR system 9600 (Perkin Elmer). After PCR amplification, 5 μl of the reaction product was analysed via gel electrophoresis and ethidium bromide staining. The remaining volume (20 μl) was purified using a Sephaglas Bandprep Kit (Pharmacia Biotech) and eluted into a final volume of 12–15 μl TE.

### Cycle sequencing

Sephaglas purified PCR fragments (6 μl) were sequenced (50% in both directions) using Amplicycle Sequencing Kits (Perkin Elmer) incorporating [$^{33}$P]dATP (NEN). Primers used for PCR amplification were used to prime the sequencing reaction for each of the small exons and, as indicated in Table 1, for exon 11. Sequenced PCR fragments were analysed using standard 6% polyacrylamide/urea sequencing gels on BIO-RAD apparatuses. Samples were loaded into 48-well combs, typically 12 fragments per gel, with all 12 A, C, G and T tracks running alongside each other. Gels were run for 3–6 h before being dried on a slab drier (Bio-Rad) (without fixation) and exposed to overnight autoradiography. The normal *BRCA1* sequence was identified, which made up the background sequencing pattern on the autoradiograph. *BRCA1* sequence variants were easily identified as they appeared as aberrant banding patterns on the normal sequence background. Fragments containing aberrant sequences were reamplified from stock DNA of the same individual and the analysis was repeated. Where possible, individuals identified to be carrying *BRCA1* mutations were bled again and the sequence analysis was repeated for a third time. Sequence variants identified as truncating mutations within exon 11 were further analysed using the protein truncation test (PTT).

### Protein truncation test (PTT)

The PTT within exon 11 was a modification of that described by Roest et al (1993), including (Seg 3 T7 and Seg 4 T7) primers described by Hogervorst et al (1995) and reverse primers described by Friedman et al (1994) using genomic DNA as the PCR template. Exon 11 was PCR amplified in three overlapping fragments. PCR fragments (250–500 ng) were then subjected to a transcription and translation protocol incorporating [$^{35}$S]methionine (Amersham) and utilizing T7 RNA polymerase, a rabbit reticulocyte lysate and a luciferase control (Promega). An additional *BRCA1* control individual was selected who did not contain a truncating mutation in exon 11 (as assessed by sequencing). Reactions were analysed via 14% sodium dodecyl sulphate polyacrylamide gel electrophoresis (SDS-PAGE) on a mini-protean II apparatus (Bio-Rad). Dried gels were exposed to overnight autoradiography, enhanced by Amplify fluorographic reagent (Amersham).

### Statistical methods

The proportion ($P$) of cases in the population who carry a germline *BRCA1* mutation/variant/polymorphism was estimated from the sequencing of $N_1$ cases with, and $N_2$ cases without, a family history, by $P = p_1n_1/N_1 + p_2n_2/N_2$ where $n_1$ and $n_2$ are the observed numbers of mutations/variants/polymorphisms and $p_1 = 0.3$ and $p_2 = 1 - p_1 = 0.7$ are the estimated proportions of all cases in the population with and without a family history, respectively, based on the total sample of 467 cases (McCredie et al, 1998). A 95% confidence interval (supported range) for $P$ was calculated from the likelihood profile (Clayton and Hills, 1993). The difference between the proportion of cases and the proportion of controls with a given polymorphism was assessed by the likelihood ratio criterion.

### RESULTS

Demographic and lifestyle characteristics of the cases and controls from which subjects were chosen for *BRCA1* sequencing are given in McCredie et al (1998). The main risk factors for early onset breast cancer are age and having a family history of breast cancer, and sampling was stratified according to the latter factor. There was no difference between sequenced and non-sequenced cases in mean age (35.0 vs. 34.8 years; $P = 0.7$), or in other established or putative risk factors for breast cancer measured in our study (McCredie et al, 1998) including parity, height, weight, age at

© Cancer Research Campaign 1999

**Table 3** *BRCA1* variants detected

| DNA variant[a] | Nucleotide change | Codon | Amino acid change | Allele frequency[b] (no. of alleles) | |
|---|---|---|---|---|---|
| | | | | Cases (91) | Controls (67) |
| Protein truncating mutations | | | | | |
| 1876 | Del C | 586 | Stop 587 | 0.003 (1) | – |
| 3888 | Del GA | 1257 | Stop 1265 | 0.008 (1) | – |
| 3415 | Del C | 1099 | Stop 1108 | 0.008 (1) | – |
| Rare variants | | | | | |
| 172 bp | T>A | 17 | Met>Lys | 0.003 (1) | – |
| 49 5′ exon 4 | C>T | – | – | 0.016 (2) | 0.030 (4) |
| 2 5′ exon 10 | A>C | – | – | 0.003 (1) | – |
| 760 | A>G | 213 | Asp>Gly | 0.003 (1) | – |
| 3238 | G>A | 1039 | Ser>Asn | 0.011 (2) | – |
| 4654 | A>T | 1845 | Ser>Ile | 0.003 (1) | – |
| 5075 | G>A | 1652 | Met>Ile | 0.019 (3) | 0.007 (1) |
| Common polymorphisms | | | | | |
| 34 5′ exon 8 | C>T | – | – | 0.30 (52) | 0.23 (31) |
| 58 5′ exon 9 | Del 1 | – | – | 0.21 (53) | 0.29 (39) |
| 1186 | A>G | 356 | Gln>Arg | 0.07 (12) | 0.04 (6) |
| 2201 | C>T | 694 | Ser>Ser | 0.21 (40) | 0.31 (42) |
| 2430 | T>C | 771 | Leu>Leu | 0.21 (40) | 0.31 (42) |
| 2731 | C>T | 871 | Pro>Leu | 0.20 (39) | 0.30 (40) |
| 3232 | A>G | 1038 | Glu>Gly | 0.13 (24) | 0.32 (43) |
| 3667 | A>G | 1183 | Lys>Arg | 0.20 (38) | 0.31 (42) |
| 4427 | T>C | 1436 | Ser>Ser | 0.21 (39) | 0.31 (42) |
| 4956 | A>G | 1613 | Ser>Gly | 0.21 (40) | 0.31 (42) |

[a]Sequence as per Genbank Accession No. U14680. [b]Adjusted for stratified sampling.

menarche and use of oral contraceptives. The percentage of women born in Australia did not differ between cases sequenced and controls sequenced (73% vs. 85%; $P = 0.06$).

### Mutations

Table 2 shows that three mutations were detected by sequencing, and confirmed to be protein-truncating by PTT analysis. They were: (i) a 1876delC mutation, which terminates translation at codon 587; (ii) a 3888delGA mutation, which terminates translation at codon 1265; and (iii) a 3415delC mutation, which terminates translation at codon 1108. All three mutations were in exon 11.

The 1876delC mutation was found in a case diagnosed in her late 30s who had a family history of breast cancer. Her mother had verified breast cancer diagnosed in her late 40s, and it was reported, but not verified, that her mother's brother had prostate cancer. A peripheral blood sample had been collected from the mother at recruitment. Sequencing of *BRCA1* showed, however, that she did not carry the 1876delC mutation. No DNA was available from the biological father whose identity, and hence family cancer history, was unknown.

The 3888delGA mutation was detected in a woman diagnosed in her 30s, who had no relative with any cancer on either side of her family. Her sister was in her early 30s and unaffected. Her mother and an aunt were alive and in their 50s, and her father and an uncle were both alive and in their 60s. One grandmother died in her 50s and the other was alive in her 90s, whereas one grandfather lived to his 80s and the other to his late 60s.

The 3415delC mutation was detected in a woman diagnosed in her 30s, who also had no relative with any cancer on either side of her family. Her sister was in her early 30s and unaffected. Her mother, father, four aunts and two uncles were alive and in their 50s and 60s. One grandmother died in her 50s (cause unknown) and the other in her 80s, whereas one grandfather died in his 30s and the other in his 50s.

In our study, 30% of all cases diagnosed before the age of 40 reported a family history in a first- or second-degree relative (McCredie et al, 1998). Therefore, taking into account the stratified sampling (Table 2), we estimated that the proportion of Australian women with breast cancer diagnosed before the age of 40 who carry a germline protein-truncating mutation in *BRCA1* is 3.8%, with a 95% confidence interval of 0.3–12.6%.

### Rare variants

Table 3 shows that seven rare variants were observed in a total of 11 cases, and two of these variants were also observed in controls.

A T→A variation at nucleotide 172 in exon 2, resulting in a Met→Lys amino acid change, was observed in a case diagnosed in her 30s, whose mother had verified breast cancer diagnosed in her early 50s. No DNA was available from her mother, but as her father did not possess the variant yet one of her sisters did it is implied that the mother also had the variant (given that paternity has been correctly reported).

An intronic C→T variation 49 bp 5′ of exon 4 was observed in two cases without a family history, and in four controls.

An intronic A→C variation 2 bp 5′ of exon 10, and an A→G variation at nucleotide 760 in exon 10, that results in an Asp→Gly amino acid change, were both observed in the same case. She was diagnosed at age 39, and had a family history; her mother was verified to have had breast cancer diagnosed in her late 40s, and also shared both these variants. There were no maternal aunts.

© Cancer Research Campaign 1999

A G→A variation at nucleotide 3238 in exon 11, resulting in a Ser→Asn amino acid change, was observed in two cases. One of these cases had bilateral breast cancer diagnosed in her 30s, and had a family history. It was verified that the grandmother on the mother's side had breast cancer diagnosed in her early 70s. Sequencing of germline DNA extracted from the paraffin-embedded formalin-fixed tumour block revealed that this grandmother carried the same variant. The mother also carried the variant, and was unaffected in her early 60s. The other case with this variant had no cancer family history; her mother was alive in her 60s, and her grandmothers lived to their 70s and 90s.

A G→T variation at nucleotide 4654 in exon 15, resulting in a Ser→Ile amino acid change, was observed in a case diagnosed in her 30s. Her family history consisted of an unverified report of breast cancer in the maternal grandmother in her 80s. The mother also had the variant, and was alive in her late 50s.

A G→A variation in nucleotide 5075 in exon 16, resulting in a Met>Ile amino acid change, was observed in three cases. One of these had a family history that was not associated with the variant, whereas the other two did not have a family history. The variant was also observed in one control who had a family history; her maternal aunt had verified breast cancer in her 70s.

### Polymorphisms

A total of 12 common variants (polymorphisms) were observed, two being intronic; see Table 3. For no polymorphism was the allele frequency in cases greater than in controls, at the nominal significance level of 0.05.

### A case with three rare *BRCA1* variants

One case had three rare variants (A→C 2 bp 5′ of exon 10, A→G at 760 in exon 10 and G→A at 5075 in exon 16). She shared the A→C and A→G variants with her mother, who had breast cancer, and the G→A variant only with her father.

### DISCUSSION

Although focused on women with breast cancer diagnosed at a young age, and despite a full sequencing of the *BRCA1* coding region, we found only a few mutations that could be unequivocally considered as deleterious (and we confirmed that they were protein-truncating). This observation is in accord with those of Langston et al (1996) and Newman et al (1998), who also found low estimates of the population prevalence of *BRCA1* mutations in early-onset cases. As well as the common polymorphisms seen in samples of women from multiple-case families and control groups (Durocher et al, 1996), our sequencing of cases revealed seven rare variants with either an unknown, or at most small, influence on susceptibility to early-onset breast cancer.

Interpreting the clinical significance of variants in the coding region of *BRCA1* is not always straightforward. First, protein-truncating mutations are presumed to be deleterious, based on functional considerations, and on the observation that these types of mutations segregate with the disease in families containing multiple cases of breast and/or ovarian cancer (Shattuck-Eidens et al, 1995). It is usually considered that if a protein-truncating mutation is observed in a case, then it is ‘the’ cause of the cancer. On a population basis, however, the percentage of such cases attributable to other causes is not zero, and, although small, it increases with age at diagnosis.

Second, polymorphisms (variants with no obvious functional effect, or that appear reasonably often with a similar frequency in cases as in controls) are not considered to have a major influence on cancer risk. Small effects (such as relative risks less than 2) can only be refuted by large population-based studies (see Bishop and Hopper, 1997).

Finally, those rare variants, or missense mutations, that are not obviously deleterious are very difficult to interpret. Extremely large population-based studies may be needed to exclude a modest effect on disease risk. Even if a rare variant is observed in controls, it may still have an effect on cancer risk (Bishop and Hopper, 1997). And, even if it is observed to track with disease within a family, it cannot necessarily be presumed to be of aetiological significance; although rare in the population it will be common within that family. Nevertheless, the proportion of cancer in the population attributable to rare variants is likely to be minimal. An important step in evaluating the clinical relevance of rare variants would be a functional assay utilizing eukaryotic cell systems.

In the interim, one way of trying to understand the significance of rare alleles and putative mutations is to pool data from comparable studies and conduct meta-analyses. One step in this direction is for researchers to report identified variants to a central registry, such as the Breast Cancer Information Core (BIC) on the World Wide Web (http://www.nhgri.nih.gov/Intramural_research/Lab_transfer/bic). None of the three protein-truncating mutations we observed have yet been reported to the BIC. Each of the three rare variants not found on the BIC was only observed in one case, and each caused an amino acid change. The two polymorphisms we observed that were not on the BIC were intronic.

Although classified in its sole report on the BIC as a possible mutation, we observed the intronic C→T variation 49 bp 5′ of exon 4 in 6% of controls and 3% of cases. Most of the five reports on the BIC of the G→A variation in nucleotide 5075, which we observed in 4% of cases and 1% of controls, considered it to be a polymorphism. The G→A variation at nucleotide 3238 in exon 11 has been reported eight times on the BIC as having unknown significance, however in one of these reports it was observed in 3% (7/242) of cases and in 4% (3/82) of controls (Durocher et al, 1996). We observed it in a case and her grandmother who had breast cancer in her 70s, but her obligate-carrier mother was unaffected in her early 60s. Therefore, none of the above three variants observed by us and others is likely to have a strong effect on early onset breast cancer risk, although a small effect cannot be categorically excluded.

The intronic A→C variation 2 bp 5′ of exon 10, described only once on the BIC and then as a possible splice-site mutation, was observed in one case and her affected mother. The case and her mother also shared the A→G variation in exon 10, but this variant has not been reported on the BIC.

Although not on the BIC, our single observation of a T→A variation at nucleotide 172, which causes a Met→Lys amino acid change, may be of interest. A T→C variation at this same site, which causes a Met→Thr amino acid change, was observed in 1 out of 80 cases and 0 out of 73 controls in a population-based study of young women with breast cancer (Langston et al, 1996). That case apparently did not have a family history. The G→T variation at 4654, seen in one case and her father, also has not been reported on the BIC.

Therefore, for none of the variants we observed is there evidence for a strong effect on breast cancer susceptibility. We observed, at most, four rare variants with any potential to have a high risk, but they are obviously very uncommon. Consequently, although we cannot discount that on an individual basis they may

*© Cancer Research Campaign 1999*

be important, on a population basis such rare alterations to *BRCA1* are of little consequence in that they are likely to explain no more than a minimal proportion of breast cancer in the population.

For the common polymorphisms, the allele frequencies we observed are consistent with those reported in other populations (Durocher et al, 1996). Given our sample sizes and the allele frequencies in controls of about 0.2–0.3, we had 80% power to detect increased risks of fourfold or more at the 0.05 level of significance for the polymorphisms. Interestingly, the allele frequencies for the exon 11 polymorphisms in pairwise linkage disequilibrium observed in control samples from Utah and Quebec are quite similar to those from our Australian sample.

The different estimates of the prevalence of mutations in *BRCA1* reported in the literature to date should not be overinterpreted. First, populations vary from one another in their racial and ethnic characteristics and origins, so one might anticipate that real differences exist both within and between populations. For example, it is already known that in the USA the Ashkenazi Jewish population has about 20 times the prevalence of mutations in *BRCA1* and *BRCA2* than thought to apply to the whole population (Struewing et al, 1997). Second, the imprecision of estimates – a consequence of the surprisingly small number of cases being found to be carriers – cannot be overlooked; see the large confidence intervals (where reported) in the Introduction and Results sections. Mutation detection, especially if it is to have close to 100% specificity (as we have attempted in this study), is very expensive and time-consuming using current technology. It is likely that this may change in the next decade (Hacia et al, 1996), so it behoves researchers to apply epidemiological rigour to the collection of large and carefully characterized population samples, paying careful attention to matching considerations (Bishop and Hopper, 1997).

The lack of family history of breast or ovarian cancer in our mutation-carrying cases, and other reports of mutation-carrying cases not necessarily having a family history of breast cancer (e.g. Langston et al, 1996), demonstrates that in the population setting a family history may be a weak predictor of *BRCA1* mutation status. Only families with an extensive history of breast and/or ovarian cancer appear to have more than a small chance of harbouring *BRCA1* mutations (Easton et al, 1993). It also raises concern about the average penetrance of those *BRCA1* mutations causing breast cancer in the population.

### ACKNOWLEDGEMENTS

We are grateful to the physicians, surgeons, oncologists and pathologists in Victoria and New South Wales who endorsed the project, to the interview and data entry staff, and to the many women and their relatives who participated in the research. This work was supported by grants from the National Health and Medical Research Council (NHMRC) of Australia, the Victorian Health Promotion Foundation, the New South Wales Cancer Council, and the Peter MacCallum Cancer Institute.

### REFERENCES

Bishop DT and Hopper JL (1997) AT-tributable risks? *Nature Genet* **15**: 226

Claus EB, Risch N and Thompson WD (1991) Genetic analysis of breast cancer in the Cancer and Steroid Hormone Study. *Am J Hum Genet* **48**: 232–242

Clayton D and Hills M (1993) *Statistical Models in Epidemiology*. Oxford: Oxford University Press

Durocher F, Shattuck-Eidens D, McClure M, Labrie F, Skolnick MH, Goldgar DE and Simard J (1996) Comparison of *BRCA1* polymorphisms, rare sequence variants and/or missense mutations in unaffected and breast/ovarian cancer populations. *Hum Mol Genet* **5**: 835–842

Easton D (1997) Breast cancer genes – what are the real risks? *Nature Genet* **16**: 210–211

Easton DF, Bishop DT, Ford D, Crockford GP and the Breast Cancer Linkage Consortium (1993) Genetic linkage analysis in familial breast and ovarian cancer: results from 214 families. *Am J Hum Genet* **52**: 678–701

Easton DF, Mathews FE, Ford D et al (1996) Cancer mortality in relatives of women with ovarian cancer: the OPCS study. *Int J Cancer* **65**: 284–294

Ford D, Easton DF, Bishop DT, Narod SA, Goldgar DE and the Breast Cancer Linkage Consortium (1994) Risks of cancer in *BRCA1*-mutation carriers. Breast Cancer Linkage Consortium. *Lancet* **343**: 692–695

Ford D, Easton DF and Peto J (1995) Estimates of the gene frequency of *BRCA1* and its contribution to breast and ovarian cancer incidence. *Am J Hum Genet* **57**: 1457–1462

Friedman LS, Ostermeyer EA, Szabo CI, Dowd P, Lynch ED, Rowell SE and King MC (1994) Confirmation of *BRCA1* by analysis of germline mutations linked to breast and ovarian cancer in ten families. *Nature Genet* **8**: 399–404

Hacia JG, Brody LC, Chee MS, Fodor SP and Collins FS (1996) Detection of heterozygous mutations in *BRCA1* using high density oligonucleotide arrays and two-colour fluorescence analysis. *Nature Genet* **14**: 441–447

Hogervorst FB, Cornelis RS, Bout M, van Vliet M, Oosterwijk JC, Olmer R, Bakker B, Klijn JG, Vasen HF and Meijers Heijboer H (1995) Rapid detection of *BRCA1* mutations by the protein truncation test. *Nature Genet* **10**: 208–212

Hopper JL and Carlin JB (1992) Familial aggregation of a disease consequent upon correlation between relatives in a risk factor measured on a continuous scale. *Am J Epidemiol* **136**: 1138–1147

Hopper JL, Giles GG, McCredie MRE and Boyle P (1994) Background, rationale and protocol for a case–control-family study of breast cancer. *Breast* **3**: 79–86

Langston AA, Malone KE, Thompson JD, Daling JR and Ostrander EA (1996) *BRCA1* mutations in a population-based sample of young women with breast cancer. *New Engl J Med* **334**: 137–142

McCredie MRE, Dite GS, Giles GG and Hopper JL (1998) Breast cancer in Australian women under the age of 40. *Cancer Causes Control* **9**: 189–198

Miki Y, Swensen J, Shattuck-Eidens D, Futreal PA, Harshman K, Tavtigian S, Liu Q, Cochran C, Bennett LM, Ding W, Bell R, Rosenthal J, Hussey C, Tran T, McClure M, Frye C, Hattier T, Phelps R, Haugenstrano A, Katcher H, Yakumo K, Gholami Z, Shaffer D, Stone S, Bayer S and Skolnick M (1994) A strong candidate for the 17q-linked breast and ovarian cancer susceptibility gene *BRCA1*. *Science* **266**: 66–71

Newman B, Mu H, Butler LM, Millikan RC, Moorman PG and King M-C (1998) Frequency of breast cancer attributable to *BRCA1* in a population-based series of European–American and African–American women. *JAMA* **279**: 915–921

Peto J, Easton DF, Mathews FE, Ford D and Swerdlow AJ (1996) Cancer mortality in relatives of women with breast cancer: the OPCS study. *Int J Cancer* **65**: 275–283

Roest PA, Roberts RG, Sugino S, van Ommen GJ and den Dunnen JT (1993) Protein truncation test (PTT) for rapid detection of translation-terminating mutations. *Hum Mol Genet* **2**: 1719–1721

Shattuck-Eidens D, McClure M, Simard J, Labrie F, Narod S, Couch F, Hoskins K, Weber B, Castilla L, Erdos M, Brody L, Friedman L, Ostermeyer E, Szabo C, King MC, Jhanwar S, Offit K, Norton L, Gilewski T, Lubin M, Osborne M, Black D, Boyd M, Steel M, Ingles S and Goldgar DE (1995) A collaborative survey of 80 mutations in the *BRCA1* breast and ovarian cancer susceptibility gene. Implications for presymptomatic testing and screening. *JAMA* **273**: 535–541

Simard J, Tonin P, Durocher F, Morgan K, Rommens J, Gingras S, Samson C, Leblanc JF, C Bl and Dion F (1994) Common origins of *BRCA1* mutations in Canadian breast and ovarian cancer families. *Nature Genet* **8**: 392–398

Southey MC, Batten LE, McCredie MRE, Giles GG, Dite G, Hopper JL and Ventor DJ (1998) Estrogen receptor polymorphism at codon 325 and risk of breast cancer before the age of 40. *J Natl Cancer Inst* **90**: 532–536

Struewing JP, Hartge P, Wacholder S, Baker SM, Berlin M, McAdams M, Timmerman MM, Brody LC and Tucker MA (1997) The risk of cancer associated with the 185delAG and 5382insC mutations of *BRCA1* and the 6174delT mutation of *BRCA2* among Ashkenazi Jews. *New Engl J Med* **336**: 1401–1408

Whittemore AS, Gong G and Itnyre J (1997) Prevalence and contribution of *BRCA1* mutations in breast cancer and ovarian cancer: results from three US population-based case–control studies of ovarian cancer. *Am J Hum Genet* **60**: 496–504

Wooster R, Bignell G, Lancaster J et al (1995) Identification of the breast cancer susceptibility gene *BRCA2*. *Nature* **378**: 789–792

© Cancer Research Campaign 1999 
